# COMISET: Dataset for the analysis of malicious events in Windows systems

**DOI:** 10.1016/j.dib.2025.111723

**Published:** 2025-05-28

**Authors:** Antonio Pérez-Sánchez, Rafael Palacios, Gregorio López López

**Affiliations:** a Institute for Research in Technology (IIT), ICAI School of Engineering, Comillas Pontifical University, 28015 Madrid, Spain; b Cybersecurity at MIT Sloan (CAMS), Massachusetts Institute of Technology, Cambridge, MA 02139, USA

**Keywords:** Event-based threat detection, MITRE ATT&CK, Cyber kill chain, Advanced persistent threats

## Abstract

The evaluation of threat detection and prevention systems requires the use of datasets that are up-to-date and correctly designed according to the most common threats. Currently, the availability of event datasets containing sufficient information to perform these analyses on Microsoft Windows systems is practically non-existent. In the background section we summarize the existing datasets, highlighting their main limitations to conduct studies of threat detection. Following we present COMISET, the dataset we have generated through the collection of events in real time and updated according to the current threats and malware obfuscation techniques. The main advantage of using this dataset with respect to those already available is that it was developed specifically for the evaluation of threat detection and prevention systems, and the events were labelled according to techniques and tactics of the MITRE ATT&CK matrix. COMISET is freely available for research purposes and contains about 250 million events of both malicious and non-malicious types. To create the dataset the experiments have been performed in two different scenarios: a laboratory emulating the infrastructure of a small company, and a computer network commonly used by students at Comillas University. In the laboratory environment, real attacks were executed involving a variety of techniques and tactics commonly used by the adversaries. The monitoring system was able to capture the events and label them according to the MITRE ATT&CK matrix. Some of these events are shown in this paper as an example of the worthy information contained in the dataset.

Specifications Table

**Table:** 

Subject	Computer Sciences		
Specific subject area	Cybersecurity		
Type of data	List of system events collected in real-time. Processed to assign MITRE labels of malicious events.		
Data collection	The information collected for the real working environment and malicious tests environment was stored in JSON format in two different files, which were obtained in two different periods of time. The dataset that corresponds to the real working environment includes the period between July 1^st^, 2022, and July 30^th^, 2022, with a size of 914GB. The dataset corresponding to the malicious test environment was collected from November 16^th^, 2022, to November 25^th^, 2022, with a size of 155GB. Table 1. summarizes the information for each of the files. Each event in JSON format found in each file has a format similar to the one below. In this case, the part belonging to an event labelled as malicious is shown.		
	Table 1. Summary of dataset information		
	Characteristics of the dataset	Real Environment	Malicious Test Environment
	
	Monitoring start	01/JUL/2022 06:26h	16/NOV/2022 08:25h
	End monitoring	30/JUL/2022 12:33h	25/NOV/2022 22:56h
	Number of events	202,304,794	49,914,325
	File size in JSON Format	914GB	155GB
	Malicious events	631,606	1,713,709
	Non-malicious events	201,673,188	482,00,616
	
	The dataset has been anonymized after data collection. This avoids displaying data that could represent a risk to the confidentiality of the organization or the users where the dataset has been collected. In order to perform these actions, an analysis of the dataset has been performed, and information has been modified, such as usernames or system names of the University by generic names. For example, user “stic” was replaced by “sxxc” and machine “aa25104pc02” was replaced by names like “computer01”. The events stored on each file contain many description details that in JSON format include the name of the field followed by the value		
Data source location	*Institute for Research in Technology (IIT), ICAI School of Engineering,Comillas Pontifical University,28015 Madrid,Spain*		
Data accessibility	*Repository name: COMISET: Dataset for the analysis of malicious events in Windows systems.* *JSON and CSV event data in a compressed file.Direct URL to data:*10.5281/zenodo.15375145		
Related research article	None		

## 1 Value of the Data

• **Realistic and Unrestricted Adversary Behaviour**: Unlike many other datasets that impose limitations on the tools or techniques used by adversaries, COMISET allows adversaries to freely choose and modify any offensive tools or malware. This flexibility results in a dataset that more accurately reflects real-world cyberattacks, where adversaries often customize their approach to bypass security defenses.
• **MITRE ATT&CK Framework Labelling**: COMISET stands out because all the collected malicious events are labelled according to the MITRE ATT&CK matrix. This standardized labelling provides a clear and structured way to analyse and understand the tactics, techniques, and procedures (TTPs) used in the dataset. Researchers can easily correlate different attack phases and techniques, facilitating a deeper understanding of adversary behaviour.
• **Multi-Stage Attack Vectors**: COMISET captures not only malware-related activities but also multi-stage attack vectors, including tactics such as lateral movement, privilege escalation, and data harvesting. This comprehensive scope makes COMISET invaluable for studying the full progression of a cyberattack, from initial compromise to exfiltration or system takeover.
• **Temporal and Event Correlation**: The dataset is designed to allow event correlation based on key identifiers, such as process IDs, parent-child relationships, and timestamps. This feature enables researchers to trace the sequence of events during an attack, providing insights into how adversaries move across a network over time and how different attack stages interconnect, making it a valuable resource for developing detection and response strategies.

## 2 Background

Threat detection and prevention systems are crucial for identifying malicious activities in networks and computer systems. These systems include Intrusion Detection Systems (IDS), antivirus (AV) software, and Endpoint Detection and Response (EDR) systems. While IDS can detect threats either at the network level (NIDS) or host level (HIDS), EDR systems offer a more comprehensive approach by combining real-time monitoring with AI algorithms.

A major challenge in threat detection is maintaining up-to-date datasets that reflect current attack techniques. Existing datasets often have limitations that hinder evaluations based on new attack scenarios. Several existing datasets only capture network traffic (MAWI [1], CTU-13 [2], UNB-ICSX-IDS-2012 [3], UNSW-NB15 [4], URG'16 [5], CIC-IDS2017 [6]) or do not include MITRE labels to categorize incidents (ADFA-LD12 [7], ADFA-WD [8], OpTC Dataset [9]). Among the most recent datasets, which include event information with MITRE labels (EVTX-ATTACK_SAMPLES [10], AAU_Maldata [11], NDSS 2024 [12]), they include Simulated-Data, or they miss the temporal component for progression analysis.

The motivation behind COMISET, a new dataset presented in this paper, is to offer a realistic resource for evaluating threat detection systems. COMISET focuses on adversary tactics and techniques as defined by the MITRE ATT&CK matrix, ensuring it reflects contemporary threats.

## 3 Data Description

The published dataset provides two files with security events generated in two different environments, a real working environment and a malicious test environment. It has been generated by monitoring real-time events from different systems over large periods of time. All the events were collected at the system level and related to the Microsoft Windows operating system. The events collected in the real working environment were generated after actions that users would normally perform. On the other hand, the events collected in the malicious test environment were generated during the execution of real malware introduced in the systems, so these events are consequences of malicious techniques applied on the system.

The information collected for the real working environment and malicious tests environment was stored in JSON format in two different files. The dataset that corresponds to the real working environment has a size of 914GB and the dataset corresponding to the malicious test environment has a size of 155GB (Table 1).

**Table 1:** COMISET data description.

Characteristics of the dataset	Real Environment	Malicious Test Environment
Number of events	202,304,794	49,914,325
File size in JSON Format	914GB	155GB
Malicious events	631,606	1,713,709
Non-malicious events	201,673,188	482,00,616

## 4 Experimental Design, Materials and Methods

### 4.1 Real-time event collection laboratories

**Fig 1:**
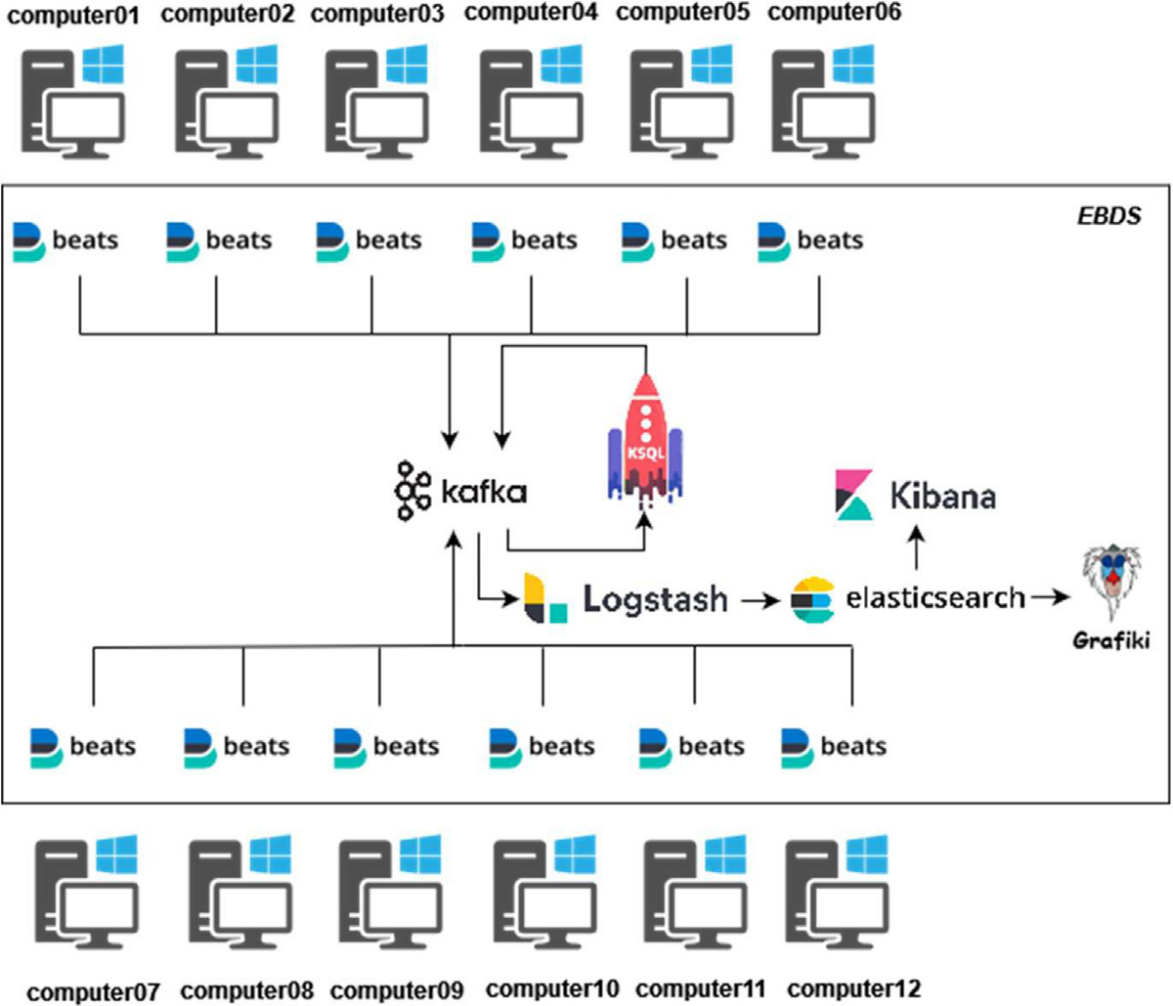
Infrastructure of the real environment for event collection.

**Fig. 2:**
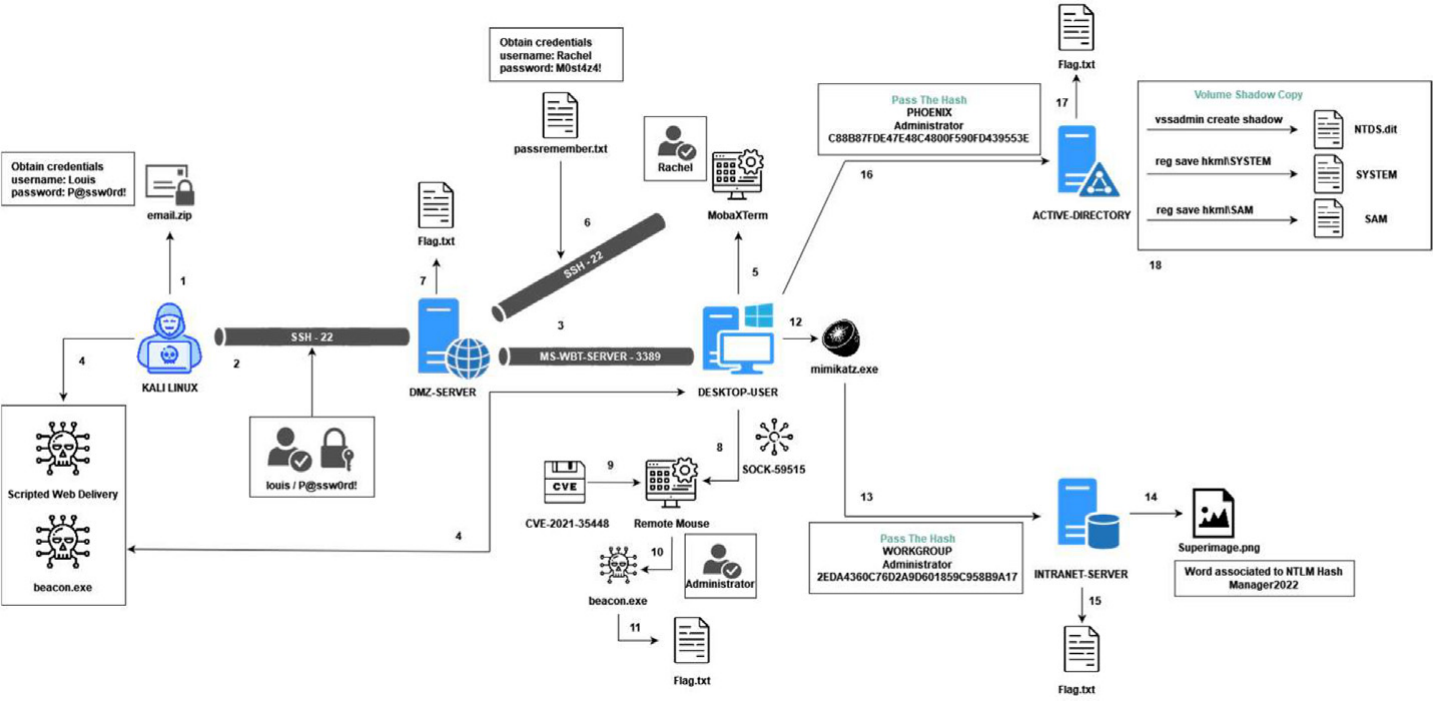
Infrastructure of the test laboratory for malicious event collection. Infrastructure components are highlighted in blue.

The COMISET dataset generation was performed with two different laboratories. These laboratories can be classified as: real working environment and malicious testing environment. In the first one, the different sensors have been installed in a computer lab at the university, and they have been monitored, collecting the events generated during a period of 1 month. These events were generated by the students in a normal working condition, without forcing any type of malicious activity, which allows us to perform a realistic analysis of events generated on a regular basis. The infrastructure monitored can be observed in Fig. 1:

The second environment is a laboratory created for the execution of offensive cybersecurity tests, and its use by different adversaries has been monitored for 3 weeks. In this case, the laboratory was prepared for the execution of different malicious techniques. As in real scenarios, each adversary is allowed to use the tactics and techniques of the MITRE ATT&CK matrix and the offensive tools that they consider necessary, as long as they achieve the objectives defined. This laboratory was used to collect events generated during active attacks that will help in future development of detection algorithms. The attacks implemented had different final objectives so red team members needed to explore network, inject malicious code, exploiting vulnerabilities in Remote Mouse process, use of Mimikatz and Pass-the-Hash to obtain admin credentials, privilege escalation, etc. Fig. 2 shows the physical infrastructure of the laboratory.

### 4.2 Architecture for malicious event detection (EBDS)

The event-based detection system was implemented by combining a set of open-source tools to automate detection of events and the transmission to cloud-based data storage. As a central core, we used Sysmon which runs as a resident service on the system and detects activity through Windows event log. The communication between systems for sending information uses Winlogbeat. This is an open-source tool for sending Windows event logs to Kafka, which is an open-source distributed event streaming platform. To centralize information, a system based on ELK (ElasticSearch, Logstah and Kibana) was also implemented. The module Logstah is a server-side data processing pipeline that ingests multiple sources simultaneously. The second module ElasticSearch provides a data search and analytics engine and sends them to Kibana that facilitates the data visualization. Finally, all system information is consumed by Grafiki, which allows to create event graphs generated during the execution of malicious techniques in the system. We have named this detection system EBDS (Event-Based Detection System) [13].

The quality of the data collected in this system can be discerned, since the base tool used for data collection is Sysmon, which runs as a resident service on the monitored system and detects activity through Windows event log. The architecture of Sysmon, complemented with the additional tools described above, has proven to effective detecting malware attacks, and helping to obtain complete graphs of techniques and tactics used by adversaries. The system was tested with a set of experiments, including evasion techniques that fooled traditional antivirus systems, through the system explained.

**Fig 3:**
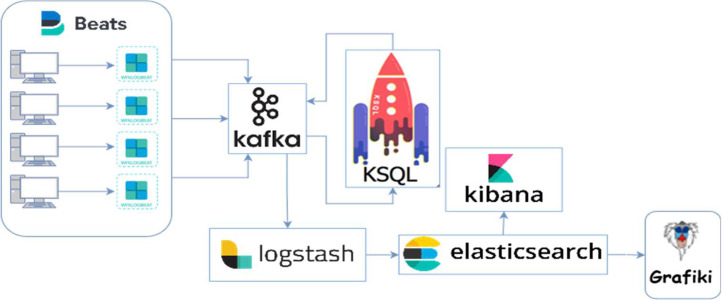
Infrastructure for malicious event detection (EBDS).

The diagram of this system can be seen in Fig. 3:

### 4.3 Labeling the events of the dataset

The process of building COMISET dataset, all the events have been labeled according to the techniques described in the MITRE ATT&CK matrix. In order to perform this labeling with the fewest possible false positives, it has been necessary to generate a large set of rules that match with the common patterns of techniques and tactics used by adversaries and create a relation between them and MITRE ATT&CK matrix. The set of rules that has been used to obtain the system events was built based on different public repositories as well as our own contributions that were not contained in the repositories. The architecture we have implemented, EBDS, can collect system events and match them with these patterns, performing the labeling in real time before storing the events. The events of the dataset incorporate some interesting fields:

• Description: Provides a description of the event or process.
• @timestamp: Indicates the timestamp of the event.
• CommandLine: This field specifies the exact command line used to start the process, including all arguments and parameters involved in its execution.
• Task: Specifies the operation associated with the event or process.
• OriginalFileName: This field identifies the original name of the executable file for the process, reflecting the filename as it was initially set during the process's execution.
• Event_recorded_time: Captures the exact timestamp when the event was recorded in the system. It provides crucial information about when the event was logged, helping to establish a timeline of activities and enabling accurate correlation with other events.
• Process_parent_id: Represents the parent Process ID (PID) of the process that initiated the current process. It helps trace the process lineage, providing insights into the hierarchical structure and execution flow of processes within the system.
• Rule_technique_name: Specifies the name of the technique from the MITRE ATT&CK framework associated with a specific rule.
• Process_parent_guid: This field provides the globally unique identifier (GUID) for the parent process that spawned the current process. It helps uniquely identify and trace the parent process across different systems and sessions, offering a precise way to map process relationships.
• Event_original_time: Captures the original timestamp when the event occurred. It provides the precise time at which the event took place, allowing for accurate timeline analysis and correlation with other activities.
• Thread_id: Represents the unique identifier (ID) assigned to the thread within a process. It helps track and distinguish between different threads running under the same process, providing insights into the execution flow and concurrency of operations.
• Current_Directory: Indicates the current working directory at the time of process execution, showing the directory path in the file system where the process runs. It provides insight into the context in which the process is operating
• Host_name: Represents the name of the host system where the event occurred. It identifies the specific machine within a network, providing context about the origin of the event and helping to distinguish between different devices.
• ParentCommandLine: Shows the command line used to launch the parent process. It includes the full command, along with any arguments and parameters, offering insight into how the parent process was started and its operational context.
• ParentUser: Denotes the user account associated with the parent process that initiated the current process. It provides insight into the user context under which the parent process was running, aiding in the identification of potential sources and levels of access involved in the event.
• process_parent_name: Specifies the name of the parent process that initiated the current process. It provides context about the originating process, helping to understand the hierarchical relationship and execution flow within the system.
• Process_id: Represents the unique Process ID (PID) assigned to the process. It helps to uniquely identify and track the specific process within the system, facilitating effective monitoring and management of process activities.
• Process_name: Indicates the name of the process being tracked. It provides the executable name or title of the process, helping to identify and distinguish it from other processes running on the system.
• Process_path: Specifies the full file path to the executable of the process. It provides the exact location within the file system where the process is stored, aiding in identifying and verifying the process's origin.
• Rule_technique_id: Contains the MITRE ATT&CK technique ID associated with the rule. It provides a unique identifier for the specific technique covered by the rule, facilitating precise mapping to the MITRE ATT&CK framework
• Process_guid: This field represents the globally unique identifier (GUID) assigned to the process. It provides a distinct reference for the process, enabling precise identification and tracking across different systems and sessions.
• User_account: Denotes the user account under which the process or event is associated. It identifies the specific user profile or credentials used, offering context about the permissions and actions performed within the system.
• Etl_host_agent_uid: Contains the unique identifier (UID) for the host agent responsible for collecting and processing event data. It helps in identifying and distinguishing between different agents within the environment, aiding in data management and troubleshooting.
• Etl_host_agent_ephemeral_uid: Provides the unique ephemeral identifier (UID) assigned to the host agent during a specific session or event. It helps track and manage the agent’s activity, particularly when the host is turned off and then turned back on, ensuring continuity in identification and monitoring
• Event_original_message: Contains the original message captured at the time the event occurred. It provides the raw, unaltered content of the event log, offering detailed context and insights into the specific event as recorded by the system.
• RuleName: This field identifies the name of the rule associated with a given event, providing key insights into the predefined criteria or conditions evaluated during the event. By mapping these rule names to the MITRE ATT&CK framework, it becomes easier to understand the specific techniques employed in the event.

### 4.4 MITRE ATT&CK heat maps generation

As a result of the analysis, the following heat maps have been obtained to show clearly which are the most used techniques of the MITRE ATT&CK matrix.

**Fig.4:**
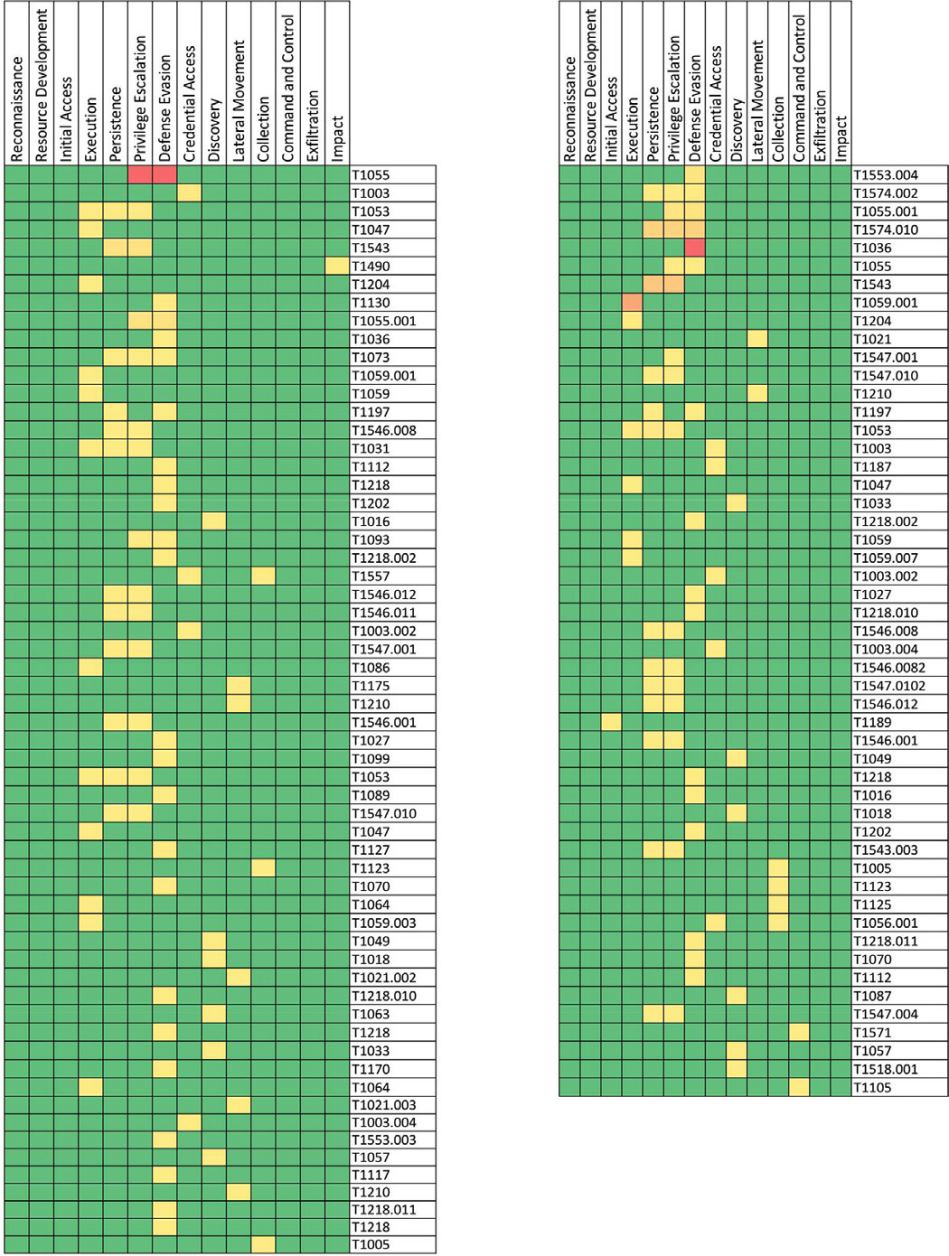
Heat map of the environments. Left: Real working environment. Right: Test laboratory environment.

The heat map corresponding to the real working environment is shown in Fig. 4 (left). As an example, it can be seen how the technique “T1055”, that corresponds to “Process Injection”, was found under the tactics “Escalation of Privileges” and “Evasion of Defences”, and they have a high volume (red) of detected events.

The heat map corresponding to the malicious testing environment is shown in Fig. 4 (right). The technique "T1036" corresponding to "Masquerading", which is part of the "Defence Evasion" tactics, has a high volume (red) of detected events.

## Limitations

Not applicable.

## Ethics Statement

The authors confirm that they have read and adhere to the ethical requirements for publication in Data in Brief. The current work does not involve human subjects, animal experiments, or any data collected from social media platforms.

## CRediT Author Statement

**Antonio Pérez-Sánchez:** Conceptualization, Methodology, Sofware, Data curation, Writing original draft. **Rafael Palacios:** Writing, reviewing and editing. **Gregorio López López:** Writing, reviewing and editing.

## Data Availability

ZenodoCOMISET: Dataset for the analysis of malicious events in Windows systems (Original data).
